# Deltamethrin and Its Nanoformulations Induce Behavioral Alteration and Toxicity in Rat Brain through Oxidative Stress and JAK2/STAT3 Signaling Pathway

**DOI:** 10.3390/toxics10060303

**Published:** 2022-06-02

**Authors:** Ahlam G. Khalifa, Walaa A. Moselhy, Hanaa M. Mohammed, Fatma Khalil, Mohamed Shaban, El-Shaymaa El-Nahass, Hessah Mohammed Al-Muzafar, Kamal Adel Amin, Khaled A. Abdou

**Affiliations:** 1 Forensic Medicine and Toxicology Department, Faculty of Veterinary Medicine, Beni-Suef University, Beni-Suef 62514, Egypt; drwalaamoselhy20@yahoo.com (W.A.M.); drkahaa20@yahoo.com (K.A.A.); 2 Cell Biology and Genetics Division, Department of Zoology, Faculty of Science, Beni-Suef University, Beni-Suef 62514, Egypt; marawanahmed20@hotmail.com; 3 Animal and Poultry Management and Wealth Development Department, Faculty of Veterinary Medicine, Beni-Suef University, Beni-Suef 62511, Egypt; fatmahs7720@yahoo.com; 4 Nanophotonics and Applications, Physics Department, Faculty of Science, Beni-Suef University, Beni-Suef 62514, Egypt; mssfadel20@aucegypt.edu; 5 Department of Pathology, Faculty of Veterinary Medicine, Beni-Suef University, Beni-Suef 62511, Egypt; shima_k8120@yahoo.com; 6 Department of Chemistry, College of Science, Imam Abdulrahman Bin Faisal University, Dammam 31441, Saudi Arabia; hmalmuzafar@iau.edu.sa; 7 Basic and Applied Scientific Research Center, Imam Abdulrahman Bin Faisal University, Dammam 31441, Saudi Arabia

**Keywords:** nanopesticides, JAK/STAT, brain, neurotoxicity, deltamethrin

## Abstract

Deltamethrin (DM) is the most powerful synthetic pyrethroid that has toxicity to the central nervous system and results in behavioral changes in both animals and humans. This effect is mediated by inducing alterations in the action of neurotransmitters and brain pathological changes. Nanocarrier encapsulated pesticides may decrease the toxicity of pesticides. Thus, this study aimed to determine the effect of an inorganic metal carrier (silica Nps) and polymeric capsule (chitosan Nps) of deltamethrin nano-formulations on antioxidant levels and oxidative stress in the brain and on behavior of the male albino rat. Sixty male albino rats were equally divided into four groups. Group I: control group; group II given DM liquefied in corn oil at 3.855 mg/kg BW; group III receiving silica-loaded deltamethrin (S/DM Nps) at 8.795 mg/kg BW; and group IV: given chitosan encapsulated deltamethrin (CS/DM Nps) at 30.44 mg/kg BW. All treatments were given orally for four weeks. Following this, behavioral tests were conducted to record locomotor activity, anxiety like behaviors, exploration, and the short memory of rats. In addition, brain antioxidant/oxidant, serum neurotransmitters such as acetylcholine esterase (AchE) and monoamine oxidase (MAO), JAK2 and STAT3 gene and proteins expression were measured. The DM group showed a highly significant elevation in malondialdehyde content, MAO, AchE, vascular endothelial growth factor (VEGF) levels, and the expression level of neurogenic genes, JAK2 and STAT3, in comparison with the control group. Both S/DM Nps and CS/DM Nps significantly decreased MAO, AchE, and VEGF compared with the DM group. Moreover, both S/DM Nps and CS/DM Nps significantly decreased the gene and proteins expression of JAK2 and STAT3 compared with the DM group. These alterations were evidenced by the deficiency in memory and learning behaviors that were accompanied by histopathological findings of the hippocampus and the cortex. It was concluded that the nano formulations containing DM induced less neurobehavioral toxicity than free DM. Additionally, the use of nanocarriers reduced the damage to health and the environment.

## 1. Introduction

Pesticides are important tools in agriculture. It is expected that their use will continue unless their detrimental effects on non-target organisms become clear [1]. The repeated and random applications of pesticide components resulted in an increase in the residual amount in the air (creating air-borne diseases), an increase in the water residual part, and the risks of eco-toxicity [2].

Pyrethroids are one of the top classes of pesticides sold, and the most important type that is easily available to the general consumer market. Exposure to pyrethroids was increased directly or indirectly as they had been substituted for organophosphorus insecticides [3]. Hence, exposure to deltamethrin (DM) has become widespread [4]. The brain is the organ most intoxicated by pesticides [5]. Among the synthetic pyrethroids, deltamethrin is the most powerful one that has both central nervous system effects and little peripheral effects [6].

DM is the most commonly used Type-II pyrethroid in mosquito control [7]. Animals and humans are exposed to DM mainly through the oral route via contaminated water and food [8]. DM exposure produces behavioral and neurochemical alterations like learning disabilities, Parkinson’s disease, birth defects, and Alzheimer’s disease [4]. DM blocks nerve impulses by modifying the kinetics of Na^+^ channels, excitatory glutamate receptors, nicotinic acetylcholine receptors, and GABA receptors [9]. The neurotoxicity of DM is mainly through the generation of free radicals, the attack of DNA, and action on neurotransmitters [10].

Toxicological reports are essential because NPs can modify the ultimate fate of the pesticides in the environment. The toxicological studies concerned with nano pesticides are still in their infancy [11]. Within the application of pyrethroid in the nanoscale form, risk assessments must be estimated along with the impact on surface water and groundwater contamination [12].

There are 3500 pyrethroid and pyrethrin products that are licensed in the United States, many of which are encapsulated formulations and are consumed worldwide. Contamination by these formulations in surface water and residues on consumed crops are plausible [13]. However, the toxicological impact is not properly studied.

In our early report we described a novel loading scheme for deltamethrin to produce chitosan NPs loaded deltamethrin (CS/DM Nps) and silica NPs loaded deltamethrin (S/DM Nps). A dynamic light scattering approach evaluated the hydrodynamic size of (S/DM Nps) and (CS/DM Nps) (284.6 ± 11.23 nm and 568.3 ± 15.36 nm, respectively) and the negative Zeta potential value (−20.8 mV and −12.5 mV, respectively). Furthermore, the larvicidal efficiency of the nanoformulations were tested using a 24 h mosquito bioassay. Our results indicated that S/DM Nps had enhanced efficacy at lower concentrations than that of DM, and that CS/DM Nps are less effective than S/DM Nps, but slow the release of DM and preserve it from early degradation [14].

There are scarce studies that have been done on the subchronic neurotoxicity of nano pesticides. Therefore, the present work was undertaken to understand the relative toxicological differences of these novel nanocarrier encapsulated DMs to determine the effect on antioxidants and oxidative stress of the brain and behavior in the male albino rat, including working memory, anxiety, locomotor, and exploratory behavior. We investigated the implication of the JAK2/STAT3 pathway in the neurotoxicity of DM and its nanometric forms.

## 2. Materials and Methods

### 2.1. Preparation of Chitosan Nps

Chitosan nanoparticles were prepared by using the ionic gelation method of sodium tripolyphosphate (TPP) with chitosan [15]. Chitosan was liquefied at 0.6% (*w*/*v*) with 1% (*v*/*v*) acetic acid. The solution was elevated to pH 4.6–4.8 with 1N NaOH. It was filtered through a 0.45 membrane (Millipore). TPP was dissolved in deionized water to 1.5% *wt*/*v* and 2 mL of TPP solution was then added drop-wise (0.3 mL/min) to 5 mL of chitosan solution with magnetic stirring at 800 rpm at room temperature for 60 min, resulting in the production of the chitosan Nps.

Nano-particles were separated by centrifugation for 30 min at 8050× *g* to remove unreacted chitosan. Distilled water was used for rinsing chitosan nanoparticles to get rid of any sodium hydroxide. The synthesized chitosan nanoparticles were kept at 4 °C and characterized [16].

### 2.2. Preparation of Deltamethrin Loaded Chitosan Nanoparticles

Deltamethrin loading was prepared by the method reported by Servat-Medina et al. [17].

### 2.3. Preparation of Silica Nanoparticles

Silica nanoparticles were prepared by tetraethyl orthosilicate hydrolysis in ethanol and ammonium hydroxide was used as a catalyst for condensation [18].

### 2.4. Loading Silica Nanoparticles with Deltamethrin

Deltamethrin loading was done using the immersion loading technique reported by Wen et al. [19]. 2.0 g of deltamethrin dissolved in 5 mL of acetone and 500 mg of silica Nps were added. The mixture was then constantly stirred at room temperature by a magnetic follower with a speed of 400 rpm. The process continued for 30 min to achieve maximum drug loading, resulting in a white turbid suspension. The material was washed with 30% ethanol and centrifuged at 13,000× *g*, 5 °C for 10 min. The end product is a white powder that has been pounded in a ceramic pestle and mortar after being dried in the air at 40 °C for 24 h.

### 2.5. Characterization of the Nanoparticles Using XRD Method

An X-ray diffractometer (XRD) was used to measure the pattern of density and size of the NPs powder. XRD analysis using Cu Kα radiation was in the 2θ range of 5–70 at a scanning speed of 5°/min (operated at 20 mA and 40 kV).

### 2.6. The Subchronic Toxicity Study of Deltamethrin, S/DM Nps, and CS/DM Nps

Sixty male albino rats, weighing 155–175 g, were kept under standard conditions. All procedures involving rats were carried out in accordance with the Animal Ethics Committee of Beni-Suef University’s Zoology Department’s rules for the care and use of experimental animals (approval number is 021-137). After two weeks of acclimation, rats were randomly grouped into four equal groups. Group I was given corn oil (1 mL/kg BW) and acted as a control group. Group II was administered DM at a dose of 3.855 mg/kg BW (corresponding to 1/10th of the median lethal dose (LD50) value: 38.55 mg/kg). Group III received S/DM Nps at a dose of 8.795 mg/kg BW (corresponding to 1/10th of LD50 value: 87.95 mg/kg). Group IV received CS/DM Nps at a dose of 30.44 mg/kg BW (corresponding to 1/10th LD50 value: 304.438 mg/kg). All treatments were given by oral gavage five days per week for 30 days. The animals were sacrificed for subsequent measurements 24 h after the final dosage. LD50 values were previously estimated [14].

### 2.7. The Effect of Deltamethrin, S/DM Nps, and CS/DM Nps on Bbehavior and Memory of Rats

#### 2.7.1. Open Field Test

The open-field test is used to measure locomotion, anxiety, and exploratory behaviors, according to Gould et al. [20]. The open field maze is constructed of wood, and its floor was divided into sixteen squares according to Brown et al. [21].

The maze was cleaned before the placement of each rat using 70% ethyl alcohol. Six rats from each group were individually placed into a corner of the maze and their behavior was video-recorded for 5 min. The behavior of each rat included locomotion (the number of peripheral squares that rats crossed with all four paws in addition to rearing; the frequency of rat stands against the maze’ walls), anxiety-like behaviors (freezing/immobility time and stretch attend posture), and exploration (number of the center square that rat entered with all four paws and time rats spent in them), and these were measured according to Walsh and Cummins [22]; Choleris et al. [23] and Kalueff and Tuohimaa [24].

#### 2.7.2. Y-Maze Test

Y-maze test measures spatial short-term working memory [25] including spontaneous alternative behavior percent (SAP) in the three arms of the maze according to the procedure described by Baluchnejadmojarad et al. [26]. Six rats from each group were used.

### 2.8. Biochemical Analysis in Brain Homogenate

#### 2.8.1. Activity of Superoxide Dismutase (SOD)

SOD activity was evaluated. A 250 μL sample, 500 μL Tris/EDTA buffer (pH 8.0), 100 μL of 10 mM pyrogallol and 250 μL distilled water made up the reaction mixture. At 420 nm, the absorbances at zero and ten minutes were measured [27].

#### 2.8.2. Lipid Peroxidation (MDA)

Lipid peroxidation was assayed. Briefly, 250 μL of homogenate was precipitated with 75 μL trichloroacetic acid (76%) and centrifuged at 3000 rpm for ten min. The collected supernatant was mixed with 175 μL thiobarbituric acid (1.07%). After 30 min of incubation at 80 °C, 250 μL cold trichloroacetic acid (90%) was added. Absorbance of the developed pink colour was read at 532 nm [28].

#### 2.8.3. Glutathione (GSH) Content

Glutathione content was measured. In brief, a 250 μL sample was precipitated with 1 mL of a precipitating solution containing 1.67% glacial metaphosphoric acid, 30% NaCl, and 0.2% EDTA. The reaction mixture was incubated at room temperature for 5 min before being centrifuged for 5 min at 3000 rpm. The clear supernatant was mixed with 0.5 mL of 5,5′-dithiobis-(2-nitrobenzoic acid) (DTNB) reagent (0.04% DTNB in 1% sodium citrate) and 2 mL of 0.3 M disodium hydrogen phosphate, and absorbance was determined at 412 nm [29].

#### 2.8.4. Activity of Glutathione-S-Transferase

The glutathione-S-transferase activity mixture consisted of 250 μL of phosphate buffer (PH 7.3), 250 μL reduced glutathione, 250 μL 1-Chloro-2,4-dinitrobenzene (CDNB) and 250 μL of the sample. The changes in the absorbance were recorded at 430 nm [30].

#### 2.8.5. Activity of Glutathione Peroxidase (GPx)

The activity of glutathione peroxidase in the homogenate was determined. Briefly, 50 μL of the sample homogenate was mixed with 50 μL of 3.28 mM hydrogen peroxide, 350 μL Tris buffer (pH 7.6) and 50 μL of 2 mM glutathione. The mixture was incubated for 10 min at room temperature. Next, 100 μL of the precipitating solution containing 1.67% glacial metaphosphoric acid, 0.2% EDTA, and 30% NaCl were added to the mixture. The reaction tubes were centrifuged for 5 min at 3000 rpm. The clear supernatant was separated and mixed with 0.5 mL of 5,5′dithiobis-(2-nitrobenzoic acid) reagent. At 412 nm, the absorbance was measured [31].

### 2.9. RNA Isolation and Quantitative Reverse Transcription-Polymerase Chain Reaction (qRT-PCR)

A gene expression analysis of Janus kinases (JAK2) and signal transducer and activator of transcription proteins (STAT3) was performed. In brief, TRIzol reagent was used to isolate total RNA from frozen cerebrum samples. Purified RNA was calculated at 260 nm and RNA samples with 260/280 ratios ≥1.7 were chosen for reverse transcription. Formaldehyde-containing agarose gel electrophoresis was also used to ensure RNA integrity.

Reverse transcription of RNA to cDNA was done with 2 μg of RNA using the Revert AidTM First Strand cDNA Synthesis Kit (MBI Fermentas, Hanover, MD, USA). Synthesized cDNA was amplified by the SYBR Green master mix in a total volume of 20 μL using the primer set reported in Table 1. Reactions were seeded in a 96-well plate and the PCR cycles included initial denaturation at 95 °C for 3 min and 35 cycles of denaturation at 95 °C for 15 s, annealing at Tm-5 for 30 s, extension at 72 °C for 30 s, and a final step at 60 °C increased about 0.5 °C every 10 s up to 95 °C. To determine the specificity of the primers, a melting curve study was done. The obtained amplification data were analyzed by the 2-ΔΔCt method and the values were optimised to β-actin [32].

### 2.10. Western Blotting Analysis

Western blotting for brain JAK2 and STAT3 was done by using the standard method. The frozen tissues were homogenized in a RIPA buffer containing proteinase inhibitors and then centrifuged at 10,000 rpm for ten minutes. Bradford’s technique was used to determine the protein concentration in the homogenates. A 10% sodium dodecyl sulfate (SDS) polyacrylamide gel electrophoresis (PAGE) was used to separate 50 μg of proteins and then the proteins were electro-transferred to the PVDF membranes. For 60 min at room temperature, the membranes were blocked in five percent *w*/*v* skimmed milk powder in Tris-buffered saline/tween 20 (TBST). After that, the membranes were incubated overnight at 4 °C with rabbit primary antibodies for JAKs, STATs, and β-actin (Santa Cruz Biotechnology, Dallas, TX, USA) diluted 1:1000 in blocking buffer. After washing with TBST, the membranes were incubated for 60 min at room temperature with peroxidase-conjugated anti-rabbit secondary antibodies, washed, and developed with an enhanced chemiluminescence kit (Thermo Scientific, Waltham, MA, USA). Image J was used to calculate the band intensity, which was then normalized to β-actin.

### 2.11. Determination of Acetylcholinesterase

Serum levels of Acetylcholinesterase were estimated using rat ELISA kits (Rat Acetylcholinesterase (AChE) ELISA Kit Cat. No. CSB E11304r (Cusabio, technology LLC, Houston, TX 77054, USA). Briefly, the standard solution or samples (100 μL) coated the multiwall plates. The liquid was then withdrawn after 60 min of incubation at 37 °C. The biotin-antibody (100 μL) was then added, incubated at 37 °C, for one hour, and was washed five times. Later, TMB substrate reagent (90 μL) was added and incubated for 15–30 min at 37 °C. Finally, each well-received 50 μL of the stop solution. At 450 nm, the optical density was observed.

### 2.12. Determination of Monoamine Oxidase

Serum levels of monoamine oxidase were assessed using rat ELISA kits (Rat monoamine oxidase (MAO) Cat. No.MBS705537 (Sofia, Bulgaria). Briefly, the standard solution or samples (100 μL) coated the multiwall plates. The liquid was then withdrawn after 2 h of incubation at 37 °C. The biotin-antibody (100 μL) was then added, incubated at 37 °C for one hour, and washed three times. Later, HRP-avidin (100 μL) was added and incubated at 37 °C for 60 min. It was then washed five times. Later, a TMB substrate reagent (90 μL) was added and incubated at 37 °C for 15–30 min. Finally, each well-received 50 μL of the stop solution. At 450 nm, the optical density was observed.

### 2.13. Determination of Vascular Endothelial Growth Factor (VEGF)

Serum levels of VEGF were assessed using rat ELISA kits (Rat vascular endothelial growth factor (VEGF) Cat. No. SL0740Ra (Sunlong, China)). Briefly, the standard solution or samples (50 μL) coated the multiwall plates. Next, it was incubated at 37 °C for 30 min and washed five times. The HRP-Conjugate reagent (50 μL) was then added, incubated for 30 min at 37 °C, and washed five times. Chromogen Solution A (50 μL) and Chromogen Solution B (50 μL) were then added and incubated for 15 min at 37 °C. Finally, each well-received 50 μL of the stop solution. At 450 nm, the optical density was observed.

### 2.14. Histological Preparations

Sectioning was stained with hematoxylin and eosin and micropathomorphologically examined [33].

### 2.15. Statistical Analysis

The statistical analyses were performed using the Statistical Package for Social Sciences (SPSS version 20.0). Dunnett’s test was used to assess the data. Values are the mean ± SD (standard deviation) for six rats in each group. *p* < 0.05 was at the accepted significance level.

## 3. Results

### 3.1. Characterization of the Nanoparticles by Using XRD Method

**Figure 1:**
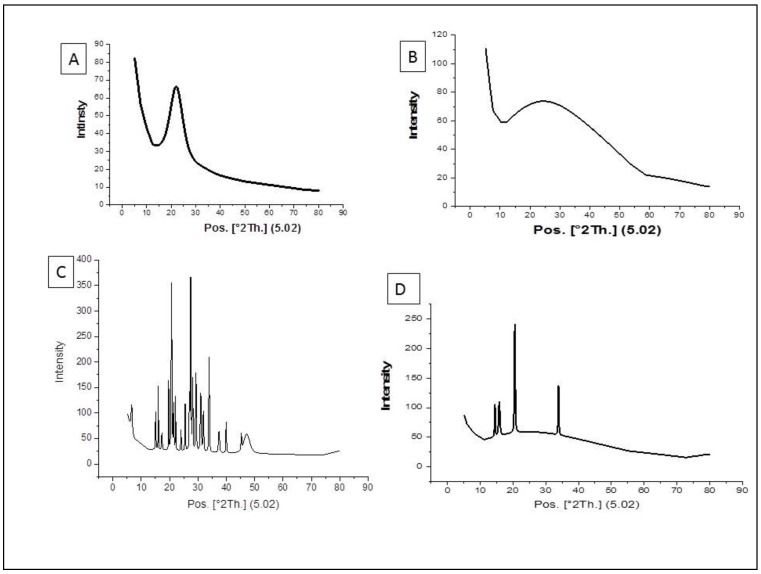
The X-ray diffraction peak of silica NPs (**A**), nano-chitosan (**B**), silica loaded deltamethrin (**C**) and chitosan loaded deltamethrin (**D**).

The result of the XRD showed the size of nano-silica, which was used in this study, was 1.095 nm, while the nano-chitosan was amorphous (Figure 1). The average size of silica-loaded deltamethrin was 38.11 nm, while that of chitosan-loaded deltamethrin was 32.44 nm (Figure 1).

### 3.2. The Effect of Deltamethrin, S/DM Nps, and CS/DM Nps on Behavior and Memory of Rats

Table 2 showed that DM reduced the activity of rats in an open field maze. The number of peripheral squares crossed by DM-treated rats was significantly (*p* = 0.01) less than that crossed by control. However, the rats performed a non-significant decrease in rearing frequency and exploratory behavior compared to control. Moreover, DM increased the anxiety-like behavior of rats (freezing time and stretch attended posture). DM significantly (*p* = 0.01) increased freezing time (second) and stretch attended posture (*p* = 0.049). Rats treated by S/DM Nps and CS/DM Nps performed behavior near to that of control groups. Furthermore, DM impaired the short-term memory of rats. Working memory (SAP) was significantly (*p* = 0.01, *p* = 0.017, *p* = 0.030) decreased in DM, S/DM Nps, and CS/DM Nps groups respectively, related to control (Table 2).

### 3.3. Antioxidant/Oxidant Level

The concentration of MDA, GSH content, and the activity of GST, GPx, and SOD in brain homogenates of experimental rats were recorded in Table 3. There were significant variations among all groups. DM caused a marked depletion (*p* < 0.05) of GSH content as well as GST and GPx stores as compared to the normal control group. The comparative level of GSH among DM and CS/DM Nps groups was not found to be significant. However, S/DM Nps did not affect GST activity compared to the control and showed an increase in GSH content compared to DM and CS/DM Nps. The GST activity was found to be lowest in the group exposed to DM, indicating the incidence of oxidative stress to be the most toxic. DM, S/DM Nps, and CS/DM Nps induced a significant decrease in the activity of SOD and a marked increase in MDA content when compared with control rats. On the other hand, there was no significance between DM and S/DM NPS or CS/DM Nps in MDA content. DM showed a more significant decrease in the activity of SOD than S/DM Nps and CS/DM Nps.

### 3.4. Acetylcholinesterase Serum Level

The analysis of the values received from the evaluation of the serum AchE level showed a highly significant increase in AchE level in the DM group, as shown in Table 4. On the other hand, no significant difference was observed between the S/DM Nps group, CS/DM Nps group, and the control group.

### 3.5. Monoamine Oxidase Level

Subchronic DM administered to rats produced a significant increase in MAO levels as compared with values of control rats and S/DM Nps. The CS/DM Nps administered groups did not show any significant change, as shown in Table 4.

### 3.6. Effect on the Serum Level of the Vascular Endothelial Growth Factor

The mean VEGF score was significantly higher in the DM group than in the control group (*p* < 0.05). No significant differences were seen among the S/DM Nps group and either the CS/DM Nps group or the control group, as illustrated in Table 4.

### 3.7. Effects of Deltamethrin, S/DM Nps, and CS/DM Nps on JAK2 and STAT3 Gene Expression

JAK2 and STAT3 mRNA abundance in the brains of deltamethrin-treated rats showed significant (*p* < 0.05) up-regulation when compared with the corresponding control as represented in Table 5. Our findings revealed an insignificant (*p* < 0.05) difference in JAK2 and STAT3 mRNA expression in S/DM Nps and CS/DM Nps administered rats. S/DM Nps and CS/DM Nps showed less of a pattern than DM.

### 3.8. Effects of Deltamethrin, S/DM Nps, and CS/DM Nps on JAK2 and STAT3 Protein Expression

**Figure 2:**
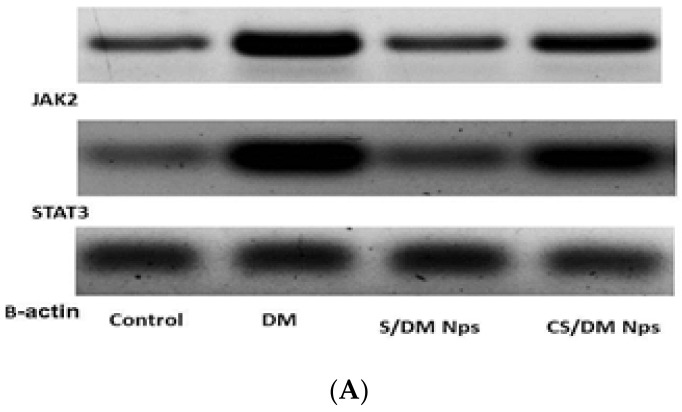

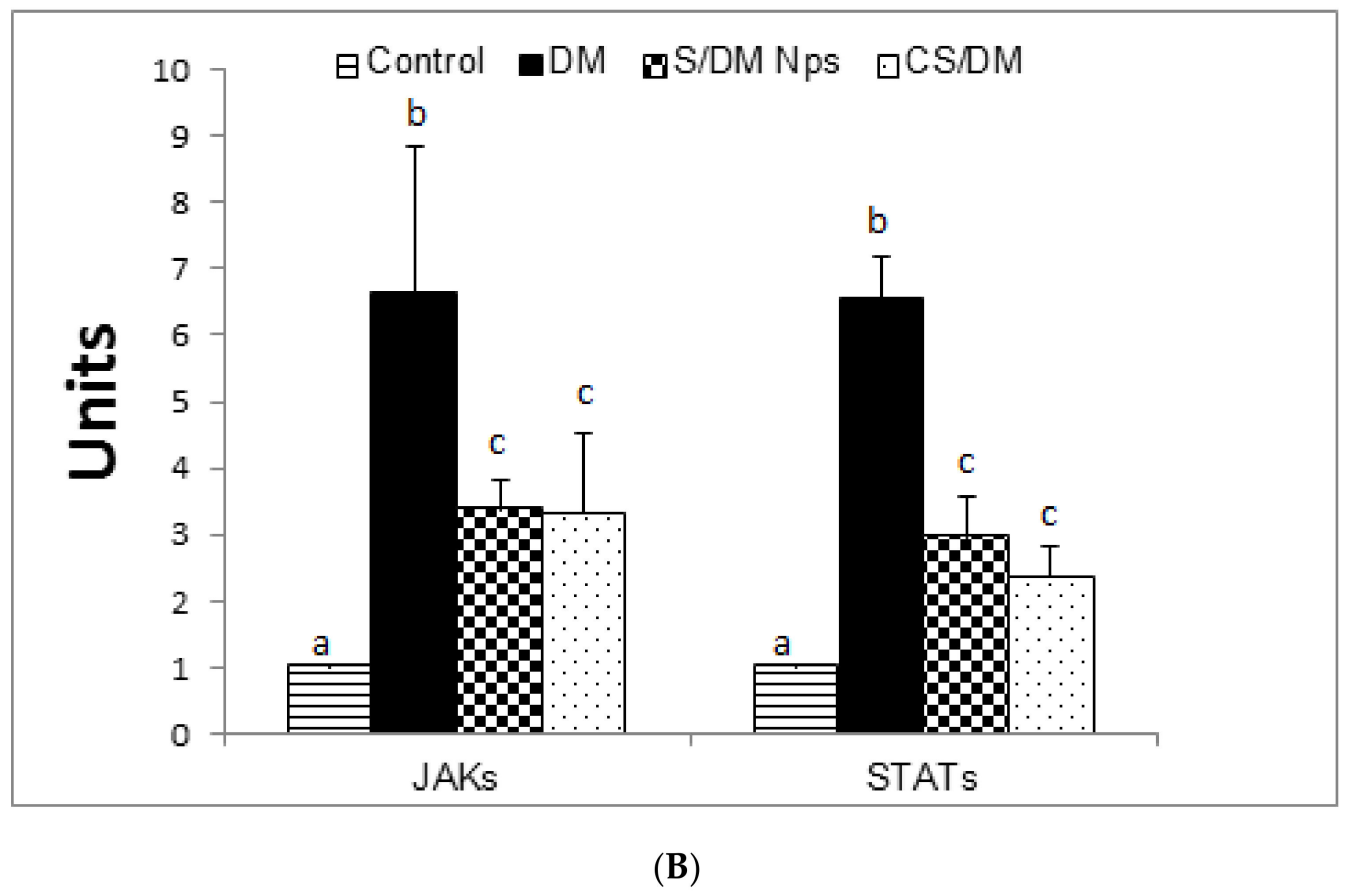
(**A**) Western blot bands of JAK2 and STAT3 respectively of deltamethrin, S/DM Nps and CS/DM Nps orally administered for 30 days. The expression of β-actin acts as loading control. (**B**) Western blot of expression pattern of JAK2 and STAT3 respectively of deltamethrin, S/DM Nps and CS/DM Nps orally administered for 30 days. Quantitative data are expressed in relative intensity arbitrary units. The bar represents the standard deviation of the mean. The different superscript letters in each group indicated a significant difference between groups at level *p* < 0.05.

Figure 2A,B and Table 6 show the expression of JAK2 and STAT3 proteins in the brains of control, deltamethrin, S/DM Nps, and CS/DM Nps treated rats after western blotting. DM treated rats showed significant (*p* < 0.05) up-regulation of JAK2 and STAT3 protein expression as compared with the control ones, while S/DM Nps and CS/DM Nps showed a significant (*p* < 0.05) decrease in the level of JAKs and STATs protein expression compared to DM treated rats.

### 3.9. Histopathological Examination

Examination of hematoxylin and eosin-stained sections revealed the presence of characteristic areas of the hippocampus and cerebral cortex.

(1) Cerebral cortex

**Figure 3:**
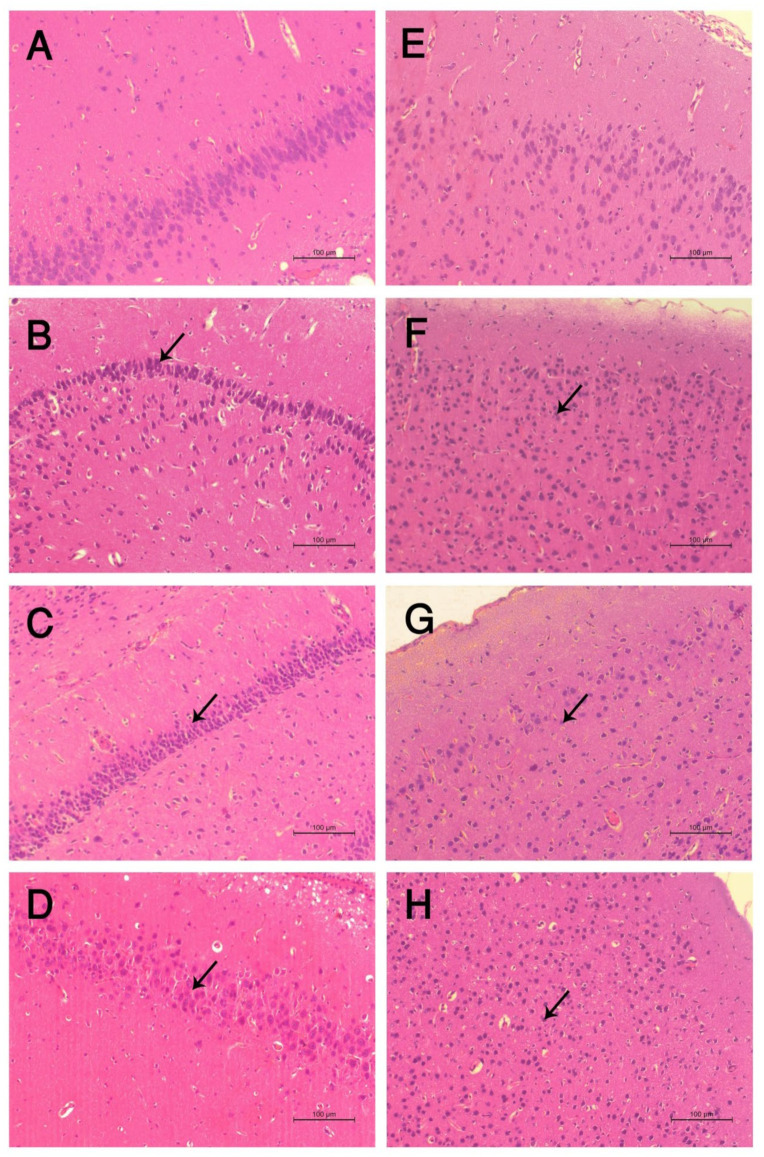
Histopathological changes in the brain of the different groups. (**A**) Hippocampus of the control group showing normal histological picture. (**B**) The hippocampus of DM treated group showing severe degenerative changes and necrosis in the pyramidal cells (arrow). (**C**) hippocampus of the S/DM Nps-treated group showing moderate degenerative changes in the pyramidal cells. (**D**) Hippocampus of the CS/DM Nps-treated group showing moderate degenerative changes in the pyramidal cells. (**E**) Cerebrum of the control group showing normal histological picture. (**F**) Cerebrum of DM treated group showing severe necrosis and degenerative changes of the neurons (arrow). (**G**) Cerebrum of S/DM Nps-treated group showing moderate degenerative changes and necrosis of neurons (arrow). (**H**) Cerebrum of the CS/DM Nps-treated group showing degenerative changes and the necrosis of neurons (arrow).

The normal structure of the cerebral cortex was shown by microscopic examination of H&E stained slides of the cerebral cortex of control negative mice. Neurons, particularly pyramidal and granule cells, as well as neuroglial cells, are common cells found inside these layers. The background was pink stained, and the neuropil was a mat of neuronal and glial cell processes. (Figure 3E). Examination of H&E stained sections taken from the DM group exhibited the presence of severe multifocal histological lesions in cerebral cortex layers as compared to the control group. These lesions were in the form of degenerative changes and the necrosis of neurons; the presence of variable sizes appeared between (extracellular) and inside most of the cells (intracellular) in all layers. Many neurons were shrunken, contained deeply stained nuclei, lost their processes, and suffered from pericellular halos. The pyramidal cells were more affected in comparison with the granular cells (Figure 3F). Sections from S/DM Nps and CS/DM Nps groups showed moderate to severe pathological changes, mainly in the form of multifocal histological lesions in the cerebral cortex layers as compared to the DM group. These lesions were in the form of degenerative changes and the necrosis of neurons (Figure 3G,H).

(2) Hippocampus

Normally, the hippocampus proper is formed by the Cornu Ammonis, which consists of cytoarchitectural fields, namely the CA1, CA2, CA3 and CA4 zones. The first and second zones consist of small, well organized pyramidal cells, while the third and fourth ones consist of large pyramidal cells. The last zone (CA4) projects into the concavity of the dentate gyrus. The pyramidal nerve cells looked like large triangular cells with large vesicular nuclei and prominent processes, especially in the CA1 zone. The control group showed a more or less normal histological structure of the previously mentioned areas (Figure 3A). Sections from the group treated with DM were markedly affected; showing disorganization and cell loss of neuronal cells in different parts. Additionally, severe degenerative changes and necrosis in the pyramidal cells (stratum pyramidale) and more shrinkage of the nuclei of cells than those of the control negative group (Figure 3B). Sections from S/DM Nps and CS/DM Nps groups showed moderate to severe pathological changes, mainly in the form of moderate degenerative changes in the pyramidal cells in CA1, CA2, CA3 and CA4 of the nuclei of cells than those of the control negative group (Figure 3C,D).

## 4. Discussion

Most of the controlled release system papers are interested in the preparation and characterization of this system, without estimation of their safety towards the environment and the living beings [11]. Nanodeltamethrin, one of such kind was encapsulated in chitosan-coated beeswax solid lipid nanoparticles (CH-BSLNs) by an association of sonication with hot homogenization. Even though NP possesses appropriate protection for DM against photodegradation [34], the toxicological research on the animal or mammalian system has yet to be performed.

Previously, we described a novel loading scheme for DM to produce chitosan loaded deltamethrin (CS/DM Nps) and silica loaded deltamethrin (S/DM Nps) with a payload percentage of 15.36% in CS/DM Nps and 37.49% in S/DM Nps. The acute toxicity of these novel nanocarriers encapsulated with DM was also studied. As reported earlier, S/DM Nps was effective and safer than the currently used DM. Enclosing DM with CS Nps sustains the release of DM and increases the release period. The estimated oral LD50 provided the acute safety of these novel DM nanoformulations [14]. This current study is complete for the safety evaluation of the nanoformulations of DM.

XRD results showed that the size of the synthesized powder is smaller than the hydrodynamic size of the nanoparticles due to their agglomeration [35]. XRD showed that the size of S/DM Nps was not as homogeneous as that of the CS/DM Nps. The homogeneous nanoparticles enhance the dissolution rates and the efficiency of a hydrophobic drug.

Reactive oxygen species are generated as an outcome of the normal metabolic processes, and their production is accelerated by accidental and occupational exposure to chemicals like pesticides. ROS are an attractive chance for the toxicity of many pesticides [36]. Of all the body organs, the brain shares more in oxidative stress [37] as it consumes a huge amount of O2 and develops a poor antioxidant network [38].

In this study, DM induced a marked and significant elevation in lipid peroxidation values in the brain homogenate of exposed animals. In addition, the activity of SOD showed a significant decrease and the concentration of GSH showed significant depletion. These results were in agreement with a study performed by Saoudi et al. [39], who reported a significant increase in MDA levels in the brains of DM- treated rats and a significant decrease in SOD, catalase, and glutathione peroxidase activities. DM appears to have a direct effect on neurons [40], and it is frequently employed as a model neurotoxicant for researching neuronal degeneration and delayed neuronal cell death, confirmed by the histopathological picture in our study [41]. The observed neurotoxicity of DM in this study was in agreement with the paper reporting that DM induces a reduction in the levels of enzymic and non-enzymic antioxidants and an increase in the amount of thiobarbituric acid reactive substances in the brain homogenate [9]. There is a strong link between oxidative damage and DM-induced neurotoxicity [38].

The central nervous system of mice was partly damaged due to oxidative stress caused by DM [42]. LPO is one of the characteristic features of the reduction of antioxidants associated with DM toxicity [43]. The brain tissue is rich in oxidizable substrates such as polyunsaturated fatty acids which are the principal goal of ROS inducing LPO [44]. The lipophilic nature of the pesticides allows them to penetrate the blood-brain barrier [45] and interact with the cellular membrane and may intensify LPO [46]. The degradation of brain lipids may be partially responsible for the neurotoxic effect of DM [47]. LPO destroys the lipid matrix of the neuronal membrane, which leads to a loss of membrane integrity; a decrease in membrane fluidity and function; inhibition of enzymes; and results in the distribution of the neuronal homeostasis, uncontrolled cellular growth, or apoptosis [48], thus leading to brain dysfunction [49]. A behavioral and physiological disturbance was observed in DM treated rats due to cell degeneration and loss of the viability of neurons in the hippocampus and striatum as intracellular ATP loss caused by LPO [50]. GST is an important mediator in pollution metabolism. [51]. GST attacks the alkyl group in the pyrethroid molecules [52] and binds it in a sequestering mechanism offering passive protection towards the pyrethroids [53]. GST is suggested to be a binding protein contributing to the action of other enzymes involved in the detoxification of pyrethroids [47]. The cytotoxic responses of deltamethrin and deltamethrin encapsulated silver nanoparticles were compared in neuronal cultured cells. Deltamethrin encapsulated silver resulted in a 17% decline in PC12 cell viability, while deltamethrin exposure caused a 47% decrease. ROS production did not significantly alter between conjugated and unconjugated ones. The toxicity of nanopermethrin was evaluated against non-target organisms. Nanopermethrin did not report antibacterial activity against *Bacillus subtilis* or against *Escherichia coli* (ATCC 13534 and 25922) [54].

In the present study, we noted that the subchronic DM administration resulted in a significant elevation in monoamine oxidase level. MAO is present on the mitochondrial membrane of presynaptic neurons. MAO is responsible for the catabolism of the amine neurotransmitters dopamine, noradrenaline, and serotonin through the oxidative removal of the amino group and regulates their extracellular concentration [55]. The S/DM Nps and CS/DM Nps administered groups did not show any significant change. High MAO activity reduced biogenic amines and increased the hydrogen peroxide level, leading to oxidative stress and cell membrane destruction and death [56]. DM increased MAO activity. This rise disrupted the neurotransmitter availability and enhanced locomotor activity and aggressive behavior [57,58].

VEGF is a potent neuroprotective molecule in vivo and in vitro experiments after diseases or injuries, as it has a direct effect on the neurons through signaling pathway activation [59]. The subchronic DM administration produced a significant increase in VEGF protein level in the serum as a response to the oxidative stress and brain injury caused by DM, while the S/DM Nps and CS/DM Nps administered groups did not show any significant change. After the oxidative stress, the VEGF expression was increased [60]. VEGF was able to stimulate angiogenesis [61] and promote the neuronal precursor’s proliferation [62]. The coordinated induction of neurogenesis and angiogenesis by VEGF could have been an adaptive value for the sustained brain repair and recovery [63]. Adult rat extraocular damaged motoneurons exhibited a noticeable choline acetyltransferase (ChAT) deregulation. VEGF administration inhibited the drop in the level of this enzyme, maintaining a normal synthesis of acetylcholine [64].

The JAK/STAT pathway is a predominant pathway that bears signals rapidly from the cell membrane to the nucleus, regulating the inflammatory cerebral response [65]. Dysregulation of the JAK-STAT pathway is linked to a number of neurodegenerative diseases, cancer, ischemia, epilepsy, angiogenesis, and inflammation of the brain [66]. DM increased the pro-inflammatory cytokine levels IL-2, IL-6, and TNF-α in rats [67]. Upon exposure to injury, microglia, immune, and glial cells release cytokines such as IL-13, IL-1, IL-6, and TNF-α [68]. These factors promote the JAK/STAT3 pathway to mediate the regional inflammatory reaction [69]. Under normal conditions, JAKs reside in the cytoplasm in an inactive form [70]. Cytokine, interferon, and interleukin attach into type I/II cytokine receptors [71]. This attachment leads to the activation of JAK proteins. Activated JAK is able to phosphorylate STATprotein by establishing docking sites for STAT. The phosphorylated STAT proteins are translocated from the cytoplasm into the nucleus [72]. At the nucleus, the activated STATs bind into DNA sequences, up-regulating the target genes [73].

The changes in GST and SOD activities indicated the formation of H_2_O_2_ [74]. The intervention of hydrogen peroxide in the brain astrocytes increased the phosphorylation levels of STAT3 and STAT1 [75]. The phosphorylated STAT is transferred to the nucleus and promotes the γ-active site of DNA, inducing the astrocytes apoptosis [76]. In the present work, we hypothesized that DM upregulated the JAK/STAT pathway through the formation of H_2_O_2_ and an increase in cytokine levels. Loading DM with silica or encapsulation with chitosan Nps appears to have a mitigating effect on the transcription responses caused by DM exposure alone.

Acetylcholinesterase (AChE) is the main enzyme responsible for the rapid hydrolytic metabolism of the neurotransmitter acetylcholine (ACh) in cholinergic pathways into choline and acetate. The decrease in the manufacture of ACh or the increase in degradation is causing disorders of memory, lack of concentration, and forgetfulness [77]. Measurement of AChE activity is one of the most widely used biomarkers for environmental medicine and pollution effects on the nervous system [78]. The principle mechanism of the neurotoxic action of pyrethroids is thought to be an effect on ion channels of the neuron membrane [40], although they may have other effects underlying the neurotoxicity. 

The results of this study report a significant increase in AchE activity in rats exposed to DM. This increase involved the reduction of the Ach, reflecting a neurobehavioral variation such as the reduction in learning efficiency and impaired memory [79]. DM administered at a dose of 0.32 mg/kg/day for 90 days showed a statistically highly significant increase in AchE activity and adrenaline in the Wistar rats hippocampus [79]. In rats, the mechanism responsible for AChE activation may be induced by adrenaline through the induction of guanylate and adenylate cyclases [80]. These findings are in agreement with several results from other papers evaluating the impact of insecticides on neurobehavioral performance in the animal model [81,82].

The obtained data indicated that DM slightly decreased the locomotion of rats in the open field. Similarly, rats orally gavaged with DM (1.0, 0.5, 0.25, and 0 mg/kg/day) from postnatal day (P) 3–20, were hypoactivated in the open field maze [58]. In addition, Nieradko-Iwanicka and Borzęcki [42] reported that IP injection of DM at doses 8.3. 20.75 and 40.5 for 28 decreased locomotor activity in mice. On the other hand, rats exposed to 5 μg of DM (i.c.v.) became hyperactive in the open field [83]. These different results may be owing to the difference in doses, routes of administration, and species. Behavioral data showed that DM induced anxiogenic effects on rats. This was expressed by the increased freezing time and stretch attend posture in the open field maze [24]. Meanwhile, anxious animals are almost less active in the open field test. Therefore, the observed decrease in the activity of rats may be because they felt anxious and not due to their inactivity in the maze. Our data revealed that DM impaired the short-term memory in rats. This result is agreeable with that reported in some early reports. Rats orally given DM (1.0, 0.5, 0.25, and 0 mg/kg/day) from postnatal day (P) 3–20, showed impairments in memory [58]. In addition, DM administration (3 mg/kg every three days for one month) caused deficits in learning and memory in mice [84].

Our findings showed that loading DM with silica or chitosan Nps could not decrease the impairment effect of DM on memory. There is a direct relationship between histopathological changes in the brain and memory retardation [84]. The obtained increase in the JAK-STAT expression indicated brain neurodegeneration [66]. Thus, the impaired memory may be attributed to brain tissue degeneration. Moreover, acetylcholine is a brain neurotransmitter included in learning processes [85]. Hence, the recorded memory deficits may be due to decreased acetylcholine levels induced by the reported increased AchE levels in our study. Furthermore, MAO inactivated neurotransmitters involved in the regulation of anxiety, locomotion, and learning [86]. Therefore, the observed increase in anxiety and learning deficits induced by DM in rats may be mediated by the reported elevation of the MAO level. 

## 5. Conclusions

This study confirms that DM, S/DM Nps, and CS/DM Nps could generate ROS. Accumulation of ROS in the brain could disturb the normal metabolism of neurotransmitters, leading to brain damage. The study revealed DM as the most toxic, followed by CS/DM Nps and S/DM Nps, as evident from the observed results. These novel nanocarrier encapsulated DM were less toxic compared to free DM, which would reduce the harm to health and increase the level of safety in applications. Further studies are highly required to investigate the involvement of the JAK2/STAT3 signaling pathway in the neurobehavioral toxicity of deltamethrin and its nanoformulations and to measure brain neurotransmitters and glial cell pathological changes.

## Figures and Tables

**Table 1:** Primers pairs used for qPCR.

Gene	GenBank Accession Number	Gene Sequence (5′-3′)
JAK2	NM_031514.1	F: GTGTGGAGATGTGCCGCTAT R: GCACTGTAGCACACTCCCTT
STAT3	NM-012747.2	F: GCAGTTTAGACAGGGAGGGG R: CACTGTCTCTGGGGCTGAAG
β-actin	NM_031144.3	F: AGGAGTACGATGAGTCCGGC R: CGCAGCTCAGTAACAGTCCG

**Table 2:** Effects of deltamethrin, S/DM Nps and CS/DM Nps sub chronic administered on locomotor, exploratory and anxiety like behaviors (in open field test) and short memory (spontaneous alternative behavior percent in a Y-maze test).

	Locomotor Behavior		Anxiety Like Behavior		Exploratory Behavior		Spontaneous Alternative Behavior Percent (SAP)
	Number of Crossed Peripheral Squares	Rearing Frequency	Freezing Time (Second)	Streched Attend Posture (Frequency)	Number of Crossed Central Squares	Time Rats Spent in Central Squares (Second)	
control	6.33 ± 6.94 ^b^	9.16 ± 5.60 ^ab^	69.33 ± 51.63 ^a^	6.00 ± 2.75 ^a^	6.00 ± 6.60 ^a^	8.33 ± 7.20 ^a^	25.83 ± 4.62 ^b^
DM	1.66 ± 1.16 ^a^	7.00 ± 3.79 ^a^	185.00 ± 47.95 ^b^	10.50 ± 3.08 ^b^	1.16 ± 1.16 ^a^	3.16 ± 5.91 ^a^	14.66 ± 5.50 ^a^
S/DM Nps	3.00 ± 2.44 ^b^	7.33 ± 1.96 ^a^	65.83 ± 19.34 ^a^	6.30 ± 4.36 ^a^	3.00 ± 2.44 ^a^	8.66 ± 8.35 ^a^	17.66 ± 3.26 ^a^
CS/DM Nps	3.83 ± 3.48 ^b^	12.16 ± 2.85 ^b^	73.50 ± 32.74 ^a^	6.00 ± 1.78 ^a^	3.83 ± 3.48 ^a^	6.00 ± 5.09 ^a^	18.33 ± 4.96 ^a^

Data are represented as mean ± standard deviation (SD) and expressed relative to control. The different superscript letters indicated a significant difference between groups at level *p* < 0.05.

**Table 3:** Effects of deltamethrin, S/DM Nps and CS/DM Nps orally administered for 30 days on the antioxidant and oxidant in brain homogenate.

Groups	Glutathione Content (nmol/ 100 mg Tissue)	Glutathione Peroxidase (mU/100 mg Tissue)	Glutathione-S-Transferase (U/100 mg Tissue)	Lipid Peroxidation (nmol MDA/100 mg Tissue/Hour)	Superoxide Dismutase (mU/100 mg Tissue)
Control	11.63 ± 1.98 ^a^	169.87 ± 6.33 ^a^	68.56 ± 11.68 ^a^	4.16 ± 0.49 ^a^	82.03 ± 2.33 ^a^
DM	4.55 ± 1.55 ^b^	54.91 ± 7.30 ^b^	24.64 ± 3.03 ^b^	7.32 ± 0.55 ^bc^	63.93 ± 3.87 ^b^
S/DM Nps	7.17 ± 1.71 ^c^	149.35 ± 4.53 ^c^	59.74 ± 6.36 ^a^	6.67 ± 1.28 ^b^	74.73 ± 2.72 ^c^
CS/DMNps	3.73 ± 2.03 ^b^	153.92 ± 12.20 ^c^	40.60 ± 4.30 ^c^	8.38 ± 1.72 ^c^	75.76 ± 1.99 ^c^

Data are represented as mean ± SD and expressed relative to control. The different superscript letters indicated a significant difference between groups at level *p* < 0.05.

**Table 4:** Effects of deltamethrin, S/DM Nps and CS/DM Nps orally administered for 30 days on the level of acetylcholinesterase, monoamine oxidase and vascular endothelial growth factor.

Groups	Acetylcholinesterase (pg/mL)	Monoamine Oxidase (mU/mL)	Vascular Endothelial Growth Factor (pg/mL)
Control	18.43 ± 2.70 ^a^	155.23 ± 3.71 ^a^	137.66 ± 4.43 ^a^
DM	42.00 ± 8.66 ^b^	215.37 ± 5.75 ^b^	324.33 ± 3.95 ^b^
S/DM Nps	27.63 ± 2.99 ^a^	177.36 ± 8.85 ^a^	94.33 ± 5.59 ^a^
CS/DM Nps	24.70 ± 7.35 ^a^	162.00 ± 7.75 ^a^	149.33 ± 6.88 ^a^

Data are represented as mean ± SD and expressed relative to control. The different superscript letters indicated a significant difference between groups at level *p* < 0.05.

**Table 5:** Effects of deltamethrin, S/DM Nps and CS/DM Nps orally administered for 30 days on JAK2 and STAT3 gene expression in the brain.

Groups	JAK2	STAT3
Control	1.01 ± 0.015 ^a^	1.00 ± 0.011 ^a^
DM	6.43 ± 1.55 ^b^	5.7 ± 0.60 ^b^
S/DM Nps	3.33 ± 0.288 ^c^	2.79 ± 0.28 ^c^
CS/DM Nps	3.46 ± 0.408 ^c^	2.23 ± 0.40 ^c^

Data are represented as mean ± SD and expressed relative to control. The different superscript letters indicated a significant difference between groups at level *p* < 0.05.

**Table 6:** Effects of deltamethrin, S/DM Nps and CS/DM Nps orally administered for 30 days on JAK2 and STAT3 protein expression in the brain.

Groups	JAK2	STAT3
Control	1.01 ± 0.01 ^a^	1.01 ± 0.015 ^a^
DM	6.66 ± 2.17 ^b^	6.57 ± 0.59 ^b^
S/DM Nps	3.37 ± 0.46 ^c^	2.96 ± 0.60 ^c^
CS/DM Nps	3.31 ± 1.22 ^c^	2.37 ± 0.46 ^c^

Data are represented as mean ± SD and expressed relative to control. The different superscript letters indicated a significant difference between groups at level *p* < 0.05.

## Data Availability

Data supporting the results during the current study are available from the corresponding author on reasonable request and all data provided in the manuscript.
